# Intracellular Bacteria Encode Inhibitory SNARE-Like Proteins

**DOI:** 10.1371/journal.pone.0007375

**Published:** 2009-10-12

**Authors:** Fabienne Paumet, Jordan Wesolowski, Alejandro Garcia-Diaz, Cedric Delevoye, Nathalie Aulner, Howard A. Shuman, Agathe Subtil, James E. Rothman

**Affiliations:** 1 Thomas Jefferson University, Department of Microbiology and Immunology, Philadelphia, Pennsylvania, United States of America; 2 Department of Cell Biology, Yale University, New Haven, Connecticut, United States of America; 3 Institut Curie, Structure et Compartiments Membranaires, CNRS-UMR144, Paris, France; 4 Institut Pasteur, Imagopole, Batiment Monod, Paris, France; 5 Department of Microbiology, Columbia University Medical Center, New York, New York, United States of America; 6 Institut Pasteur, Unité de Biologie des Interactions Cellulaires, CNRS-URA 2582, Paris, France; Duke University Medical Center, United States of America

## Abstract

Pathogens use diverse molecular machines to penetrate host cells and manipulate intracellular vesicular trafficking. Viruses employ glycoproteins, functionally and structurally similar to the SNARE proteins, to induce eukaryotic membrane fusion. Intracellular pathogens, on the other hand, need to *block* fusion of their infectious phagosomes with various endocytic compartments to escape from the degradative pathway. The molecular details concerning the mechanisms underlying this process are lacking. Using both an *in vitro* liposome fusion assay and a cellular assay, we showed that SNARE-like bacterial proteins block membrane fusion in eukaryotic cells by directly inhibiting SNARE-mediated membrane fusion. More specifically, we showed that IncA and IcmG/DotF, two SNARE-like proteins respectively expressed by *Chlamydia* and *Legionella*, inhibit the endocytic SNARE machinery. Furthermore, we identified that the SNARE-like motif present in these bacterial proteins encodes the inhibitory function. This finding suggests that SNARE-like motifs are capable of specifically manipulating membrane fusion in a wide variety of biological environments. Ultimately, this motif may have been selected during evolution because it is an efficient structural motif for modifying eukaryotic membrane fusion and thus contribute to pathogen survival.

## Introduction

In eukaryotic cells, intracellular membrane fusion events are mediated by members of the SNARE protein family. SNAREs are conserved in all eukaryotes and are present on the surface of all secretory compartments [1], [2], [3]. During membrane fusion, t-SNAREs present on target organelles assemble into a four-helix bundle with the v-SNAREs present on vesicles. This event brings the membranes in which they are embedded into close apposition and drives bilayer fusion [4], [5], [6], [7]. The SNARE residues indispensable for membrane fusion form the “SNARE motif” [6], a 60 amino-acid sequence composed of coiled-coil heptad repeats [Table 1 and [8]]. Similar structural motifs are used for the same purpose by viruses, highlighting the general role of coiled coil sequences in manipulating membrane fusion [9], [10]. Here we investigated whether this particular motif is also utilized by bacteria to influence eukaryotic membrane fusion.

**Table 1:** SNARE motifs alignment.

		...*..*...*..*...*..*...*..*...*..*...*..*...*..*...*..*....	
hSNAP25-Nterm	19	DQLADESLESTRRMLQLVEESKDAGIRTLVMLDEQGEQLERIEEGMDQINKDMKEAEKNL	
hSNAP25-Cterm	140	DARENEMDENLEQVSGIIGNLRHMALDMGNEIDTQNRQIDRIMEKADSNKTRIDEANQRA	
hSNAP23-Cterm	146	DAREDEMEENLTQVGSILGNLKDMALNIGNEIDAQNPQIKRITDKADTNRDRIDIANARA	
Sec9p-Cterm	588	DEMELEIDRNLDQIQQVSNRLKKMALTTGKELDSQQKRLNNIEESTDDLDINLHMNTNRL	
hStx1a	192	LSEIETRHSEIIKLENSIRELHDMFMDMAMLVESQGEMIDRIEYNVEHAVDYVERAVSDT	
hStx4	200	LNEISARHSEIQQLERSIRELHDIFTFLATEVEMQGEMINRIEKNILSSADYVERGQEHV	
Sso1p	190	LAEVQARHQELLKLEKSMAELTQLFNDMEELVIEQQENVDVIDKNVEDAQLDVEQGVGHT	Q-SNAREs
hStx5	209	DSYIQSRADTMQNIESTIVELGSIFQQLAHMVKEQEETIQRIDENVLGAQLDVEAAHSEI	
Sed5p	249	NVYLQERNRAVETIESTIQEVGNLFQQLASMVQEQGEVIQRIDANVDDIDLNISGAQREL	
Vam3p	190	TIIHQERSQQIGRIHTAVQEVNAIFHQLGSLVKEQGEQVTTIDENISHLHDNMQNANKQL	
hStx7	165	LRLIHERESSIRQLEADIMDINEIFKDLGMMIHEQGDVIDSIEANVENAEVHVQQANQQL	
Pep12p	195	QNLIEQRDQEISNIERGITELNEVFKDLGSVVQQQGVLVDNIEANIYTTSDNTQLASDEL	
Tlg2p	244	EAYLRERDEEITQLARGVLEVSTIFREMQDLVVDQGTIVDRIDYNLENTVVELKSADKEL	
Stx6	163	QLIVEQQDEQLELVSGSIGVLKNMSQRIGGELEEQAVMLDDFSHELESTQSRLDNVMKKL	
Tlg1p	132	EQMLREQDVHLDGIHKTMQNLHIQAQTMGDELENQGQLLDNMDEGMDGVVNKLARGRRQL	
Vam7p	250	MQMVRDQEQELVALHRIIQAQRGLALEMNEELQTQNELLTALEDDVDNTGRRLQIANKKA	
Vti1p	124	HAILQKSGDRLKDASRIANETEGIGSQIMMDLRSQRETLENARQTLFQADSYVDKSIKTL	
IcmG/DotF	146	GEQINAVNNNIKNLNAQIVNLNQIIGNMSNQIARQSEVINVLMARTTPKKVVKVSRPIVQ	
CtrIncA-Nterm	92	YQDLQREVGSLKEINFMLSVLQKEFLHLSKEFATTSKDLSAVSQDFYSCLQGFRDNYKGF	SNARE-
CtrIncA-Cterm	210	TVVIEELKTIRDSLRDEIGQLSQLSKTLTSQIALQRKESSDLCSQIRETLSSPRKSASPS	like
CcaIncA-Nterm	126	VRHMKQQIQQFGEENTRLHTAVENLKAVNVELSEQINQLKQLHTRLSDFGDRLEANTGDF	proteins
CcaIncA-Cterm	233	MSSVTELRTNLNALKELITENKTVIEQLKADAQLREEQVRFLEKRKQELEEACSTLSHSI	
hSyb1	25	PPNMTS.NRRLQQTQAQVEEVVDIIRVNVDKVLERDQKLSELDDRADALQAGASQFESSA	
hSyb2	22	PPNLTS.NRRLQQTQAQVDEVVDIMRVNVDKVLERDQKLSELDDRADALQAGASQFETSA	
hSyb3	6	TAATGS.NRRLQQTQNQVDEVVDIMRVNVDKVLERDQKLSELDDRADALQAGASQFETSA	
hVAMP8	2	EASEGGGNDRVRNLQSEVEGVKNIMTQNVERILARGENLEHLRNKTEDLEATSEHFKTTS	R-SNAREs
Snc1p	20	PQNVQS.KSRTAELQAEIDDTVGIMRDNINKVAERGERLTSIEDKADNLAVSAQGFKRGA	
Nyv1p	157	NGQNTI.SDIGDATEDQIKDVIQIMNDNIDKFLERQERVSLLVDKTSQLNSSSNKFRRKA	
Sec22p	122	SYSDKKVQDNLDQLNQELVGVKQIMSKNIEDLLYRGDSLDKMSDMSSSLKETSKRYRKSA	
mSec22b	126	YIDSRA.RRNLGSINTELQDVQRIMVANIEEVLQRGEALSALDSKANNLSSLSKKYRQDA	

SNARE motifs from yeast and mammals were aligned with C*tr*IncA-N and C-term, C*ca*IncA-N and C-term and IcmG/DotF's SNARE-like motifs (grey). The amino acids indicating the layers in the heptad repeat are highlighted in bold (asterisk). Notice the conserved glutamine and arginine residues in the central ‘d’-position of the heptad repeat, which constitute the zero layer. Stx = syntaxin. N-term and C-term refer to the N-terminal and C-terminal coiled-coil domain, respectively.

Intracellular bacteria such as *Salmonella*, *Mycobacterium*, *Legionella* or *Chlamydia* must manipulate membrane fusion of the host cells they inhabit in order to escape lysosomal fusion [11], [12]. While intracellular, these bacteria modify their infectious phagosomes, also called inclusions or vacuoles, by expressing their own proteins to the surface [13]. As a result, the infectious phagosomes become protected against fusion with endocytic compartments [14], [15]. Although the precise mechanism is unclear, it is likely that the bacterial proteins expressed on the surface of these infectious phagosomes are responsible for blocking fusion with the endocytic compartments [16], [17]. Interestingly, over the past few years a growing number of SNARE-like proteins have been identified notably in *Chlamydia* and *Legionella* [18], [19], [20], two intracellular bacteria responsible for human diseases. For instance, IncA, a protein expressed by *Chlamydia* on the surface of the infectious vacuole displays two SNARE-like motifs [19], [21]. IncA interacts directly with mammalian SNAREs [21] and IncA expressed by *Chlamydia trachomatis*, C*tr*IncA, has been implicated in homotypic membrane fusion [22], [23]. Formation of *Chlamydia* inclusions by homotypic fusion is an event specifically occurring during *C. trachomatis* infection. Interestingly, most *Chlamydia* strains express IncA, yet not all strains have the capacity to undergo homotypic fusion, suggesting that IncA likely plays additional roles.

Expressed by *Legionella pneumophila*, IcmG/DotF only displays one SNARE-like motif. The precise function of IcmG/DotF is still unclear, although mutants are rapidly trafficked to, and degraded within lysosomal compartments [24]. Using these bacterial SNARE-like proteins as our models, we tested their function on SNARE-mediated membrane fusion. We discovered a novel inhibitory function of these proteins and characterized the molecular mechanism they use to block host membrane fusion.

## Results and Discussion

Intracellular bacteria primarily protect their vacuoles against endocytic fusion [12], [25], which is mediated by the association of the v-SNARE VAMP8 with the endocytic t-SNARE composed of Syntaxin 7, Syntaxin 8 and Vti1b [26], [27].

Two distinct complementary mechanisms have been suggested concerning chlamydial avoidance of lysosomal fusion: 1) during the first ∼8 hours of infection, the protection of the vacuole appears to be independent of *Chlamydia* protein synthesis [28]. Rather, structural components of the *Chlamydia* cell wall seems to be involved in this activity [29]. 2) Later however, at a time that coincides with IncA expression [30], an active modification of the inclusion membrane takes place to sustain the protection of the inclusion. In light of these evidences, we started to investigate the role of IncA in the protection of the Chlamydia inclusion.

**Figure 1:**
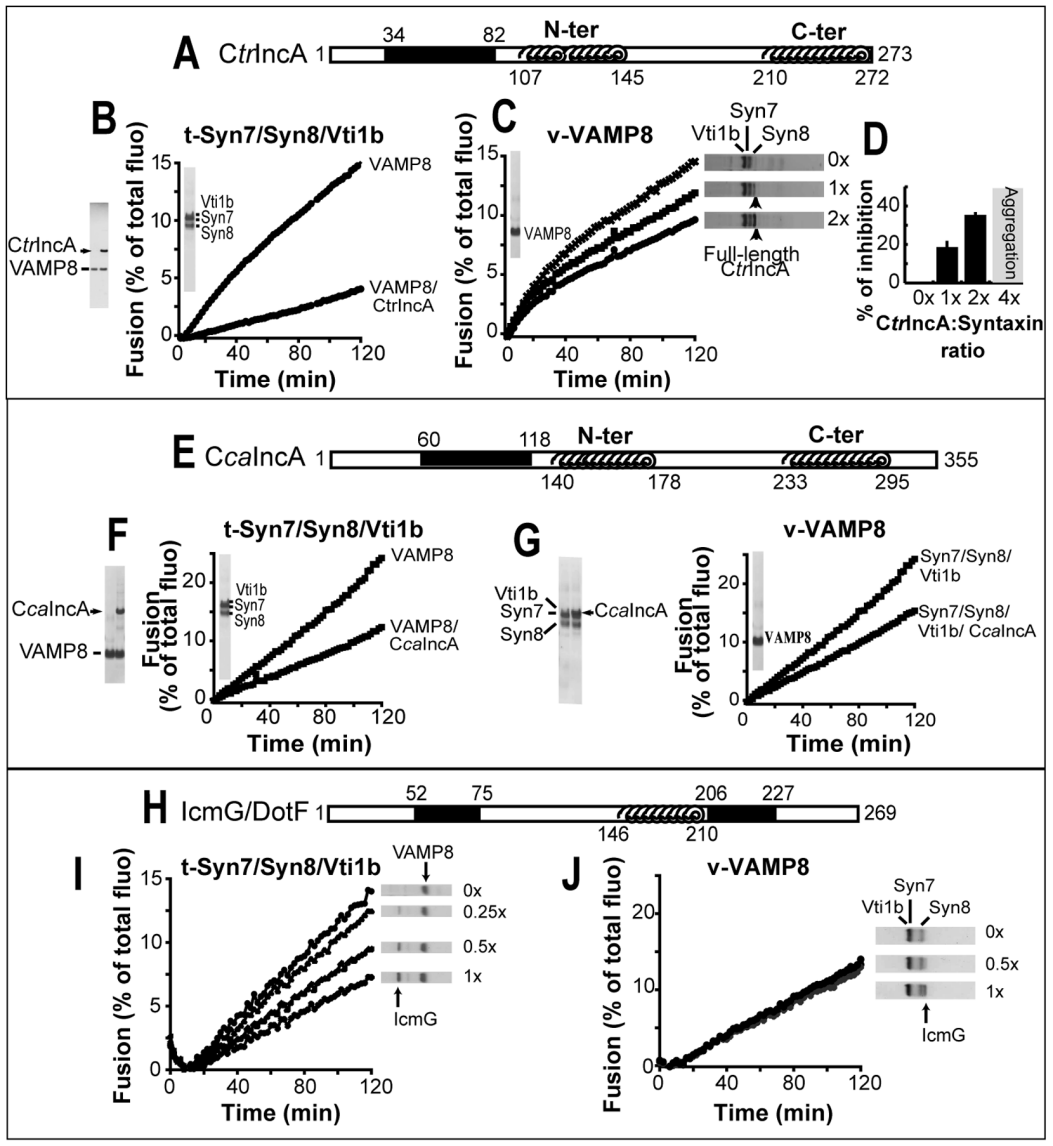
SNARE-like bacterial proteins inhibit endocytic SNARE-mediated membrane fusion. A- C*tr*IncA encodes a transmembrane domain (aa34–82), a N-terminal (N-ter: aa107–145) and a C-terminal (C-ter: aa210–272) SNARE-like motif. B- VAMP8 was reconstituted with and without C*tr*IncA into donor liposomes (coomassie gel), and incubated with t-SNARE liposomes containing [Syn7/Syn8/Vti1b]. Fusion decreases in presence of C*tr*IncA (70% inhibition). C- t-[Syn7/Syn8/Vti1b] was reconstituted with different concentration of C*tr*IncA into acceptor liposomes. Fusion shows a concentration dependency for C*tr*IncA inhibition. D- The percentage of inhibition with the standard deviation is plotted (n = 3). For each experiment, results were normalized based on the fusion rate obtained after 2 hrs with the endocytic complex w/o C*tr*IncA (0x). We observed 20% inhibition for a C*tr*IncA:Syn7 estimated *ratio* of 1:1 (1x). The inhibition rate increased to 35% for an estimated *ratio* of 2∶1 (2x). E- C*ca*IncA encodes a transmembrane domain (aa60–118), a N-terminal (N-ter: aa140–178) and a C-terminal (C-ter: aa233–295) SNARE-like motif. F- We observed 50% inhibition when C*ca*IncA was reconstituted on the v-SNARE side with VAMP8. G- We observed 40% inhibition when C*ca*IncA was reconstituted with [Syn7/Syn8/Vti1b] into acceptor liposomes. H- IcmG/DotF displays two hydrophobic regions (aa52–75 and 206–227), and a SNARE-like motif (aa146-210). I- Endocytic fusion is reduced when IcmG/DotF is present in v-SNARE liposomes and the inhibition rate correlates with IcmG/DotF concentration, reaching 50% of inhibition for an estimated *ratio* IcmG:VAMP8 of 1∶2. J- IcmG/DotF does not interfere with membrane fusion when present in t-SNARE liposomes. All graphs are representative of at least 3 independent experiments.

Previously, IncA has been shown to co-precipitate with the endocytic SNAREs when expressed in cells [21]. Using an *in vitro* liposome fusion assay [4], [6], we now tested both C*tr*IncA and C*ca*IncA expressed respectively by *C. trachomatis* and *C. caviae*, for their functional effect on endocytic SNARE-mediated membrane fusion (see Table 2 for a description of all the SNARE proteins studied here). To do so, we reconstituted the t-SNARE [Syntaxin7/Syntaxin8/Vti1b] and the v-SNARE [VAMP8] with or without IncA into acceptor and donor liposomes, respectively. Donor liposomes contain the FRET pair Rhodamine-PE [N- (lissamine rhodamine B sulfonyl) phosphatidyl ethanolamine] and NBD-PE [N- (7-nitro-2,1,3-benzoxadiazole-4-yl) phosphatidyl ethanolamine]. Liposome fusion results in lipid mixing of donor and acceptor liposomes. As the distance between NBD and rhodamine increases, the resonance energy transfer and the quenching of NBD are reduced. Fusion becomes detectable as an increased NBD fluorescence at 538nm [4], [6]. After mixing different combinations of t- and v- liposomes+/−IncA, liposome fusion was allowed to proceed at 37°C for two hours. As shown in Fig. 1, C*tr*IncA strongly inhibits endocytic SNARE-mediated fusion. C*tr*IncA blocks membrane fusion whether present in v-SNARE (∼70% inhibition) or in t-SNARE (∼37% inhibition) liposomes (Fig. 1B and 1C). We observed that the inhibitory effect of C*tr*IncA correlates with its concentration in the liposomes (Fig. 1C and 1D). Similarly, C*ca*IncA inhibits endocytic SNARE-mediated fusion whether present on t-SNARE (∼40% inhibition) or v-SNARE liposomes (∼50% inhibition) (Fig. 1G and 1F), confirming the inhibitory role of IncA proteins. When we compared both the effects of IncA and IcmG/DotF, a SNARE-like protein (Fig. 1H) expressed by *Legionella pneumophila* [Table 1 and [18]], we also observed inhibition of SNARE-mediated membrane fusion (Fig. 1). Interestingly, IcmG/DotF has no effect when present on the t-SNARE side (Fig. 1J), but only interferes with the v-SNARE (Fig. 1I). Although both IncA and IcmG/DotF have a common inhibitory function on membrane fusion, it appears that bacterial SNARE-like proteins display different levels of efficiency. Most likely, other SNARE-like proteins, such as LegC3, play a major role in protecting *Legionella*'s vacuole [31].

**Table 2:** SNARE proteins description.

SNARE investigated	Category	Location
Syntaxin7	t-SNARE	Late endosome/lysosome
Syntaxin8	t-SNARE	Late endosome/lysosome
Vti1b	t-SNARE	Late endosome/lysosome
VAMP8	v-SNARE	Late endosome/lysosome (mast cell secretory granules)
Syntaxin2	t-SNARE	Plasma membrane
Syntaxin3	t-SNARE	Plasma membrane
Syntaxin4	t-SNARE	Plasma membrane
SNAP23	t-SNARE	Plasma membrane
VAMP2	v-SNARE	Secretory vesicle

The SNAREs involved in endocytosis are Syntaxin 7, Syntaxin 8, Vti1b and VAMP8, while the SNAREs involved in exocytosis are Syntaxin 2, Syntaxin 3, Syntaxin 4, SNAP23 and VAMP2.

Although C*tr*IncA has been previously implicated in homotypic membrane fusion [22], [23], we did not observe any fusion events between C*tr*IncA-containing liposomes (data not shown). Perhaps C*tr*IncA requires post-translational modifications such as phosphorylation, to become fusogenic [32]. Consistent with this possibility, IncA has multiple phosphorylation sites that become phosphorylated by host cells during infection [19], [32]. Alternatively, additional proteins from either *Chlamydia* or the host cell might be necessary in combination with C*tr*IncA to promote fusion. Interestingly, some non-fusogenic strains do express a normal IncA protein on the inclusion membrane, supporting the possibility that other elements of the fusion machinery are missing in these strains [33].

We propose that C*tr*IncA could function as a switch to regulate the maturation of the inclusion. During the infectious cycle of *C. trachomatis*, each newly synthesized C*tr*IncA would first bind every resident SNARE on the inclusion, until all are blocked. As a consequence, SNARE-mediated fusion of the inclusion would be totally inhibited. As C*tr*IncA continues to accumulate, excess C*tr*IncA would then be available for further modification by the host cell (phosphorylation) and/or for binding additional proteins. CtrIncA would become active for fusion and inclusions could then undergo homotypic fusion.

**Figure 2:**
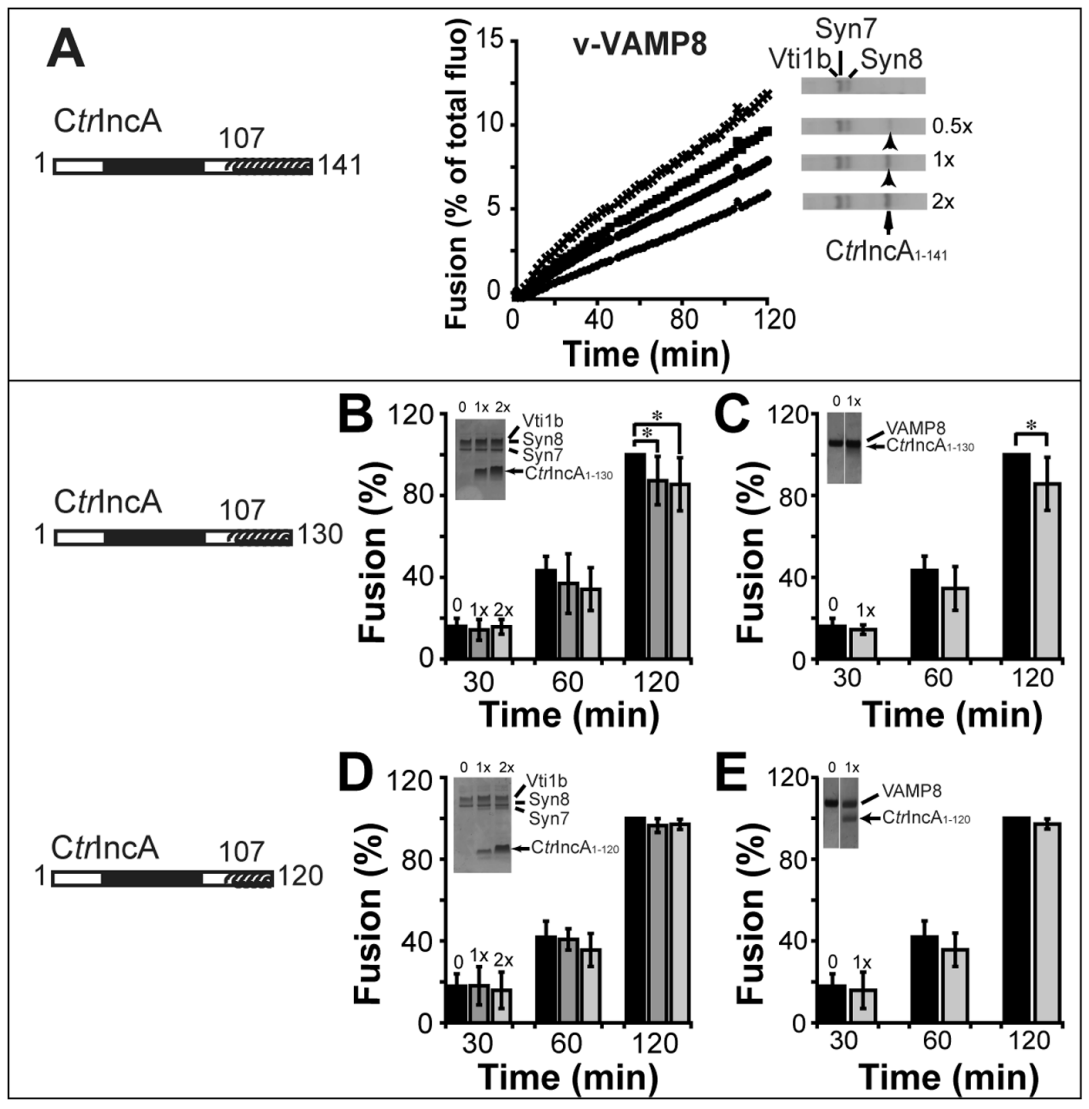
The SNARE-like motif encodes the inhibitory function. A- Increasing concentrations of C*tr*IncA_1–141_ were reconstituted into endocytic t-SNARE liposomes, and fusion proceeded in presence of VAMP8-liposomes. Fusion is significantly inhibited by the presence of C*tr*IncA_1–141_ and is dependent upon its concentration, reaching 55% of inhibition after 2 hrs with an estimated C*tr*IncA_1–141_:SNARE *ratio* of 2∶1. This experiment is representative of n = 3. B–E- Two different concentrations of truncated C*tr*IncA (see representative coomassie gels inserted in each graph) were reconstituted into t-SNARE (B,D), and v-SNARE liposomes (C,E). As shown on graphs B and C, C*tr*IncA_1–130_ still displays a significant inhibitory effect on the endocytic SNARE-mediated membrane fusion (average of 15% inhibition after 2 hrs, p = 0.022). On the contrary, C*tr*IncA_1–120_ (D,E) completely fails to inhibit endocytic fusion (p>0.05). The mean from n = 5 independent experiments was determined at 30 min, 60 min and 120 min. The standard deviation is shown. One asterisk denotes statistically significant differences (p<0.05). For the purpose of comparison, maximal values of fusion obtained for the SNARE complex without IncA at 120 min were arbitrarily defined as 100%.

Next, we determined whether the inhibitory function was encoded into the SNARE-like motif. Since IcmG/DotF has a limited inhibitory effect, we concentrated our efforts on IncA. IncA possesses two SNARE-like motifs [21] (Table 1, Fig. 1A). We focused our attention on the N-terminal motif due to its presence next to the trans-membrane domain mimicking the eukaryotic SNARE configuration. This makes it ideally located to interact directly with eukaryotic SNARE motifs. Furthermore, this motif has previously been shown to be compatible with the formation of a stable complex with SNARE proteins [19]. To determine whether the N-terminal SNARE-like motif has an inhibitory activity, truncated forms of C*tr*IncA were generated and their effects on endocytic SNARE-mediated fusion were examined. C*tr*IncA mutant containing only the N-terminal SNARE-like motif (C*tr*IncA_1–141_) inhibited endocytic SNARE-mediated fusion in a dose-dependent manner similar to the full-length protein (Fig. 2A). Next, we delineated the minimal IncA sequence necessary to retain the inhibitory function. As shown on Fig. 2B and Fig. 2C, C*tr*IncA_1–130_ still displays a significant inhibitory effect (∼15%, p = 0.028) when present on either t- or v-SNARE membrane. On the contrary, C*tr*IncA_1–120_, which contains only half of the SNARE-like motif, completely lost its ability to inhibit endocytic SNARE-mediated membrane fusion (Fig. 2D, 2E, p = 0.42). The outcome was similar regardless of whether this truncated form of IncA was reconstituted into t-SNARE or v-SNARE liposomes. This suggests that the C*tr*IncA N-terminal SNARE-like motif requires a SNARE-like motif of at least ∼23 amino acids in order to exert an effective inhibitory activity and confirm the role of this motif in blocking membrane fusion. Although the function of the C-terminal domain remains to be determined, we cannot exclude its role in reinforcing the inhibitory effect of IncA.

**Figure 3:**
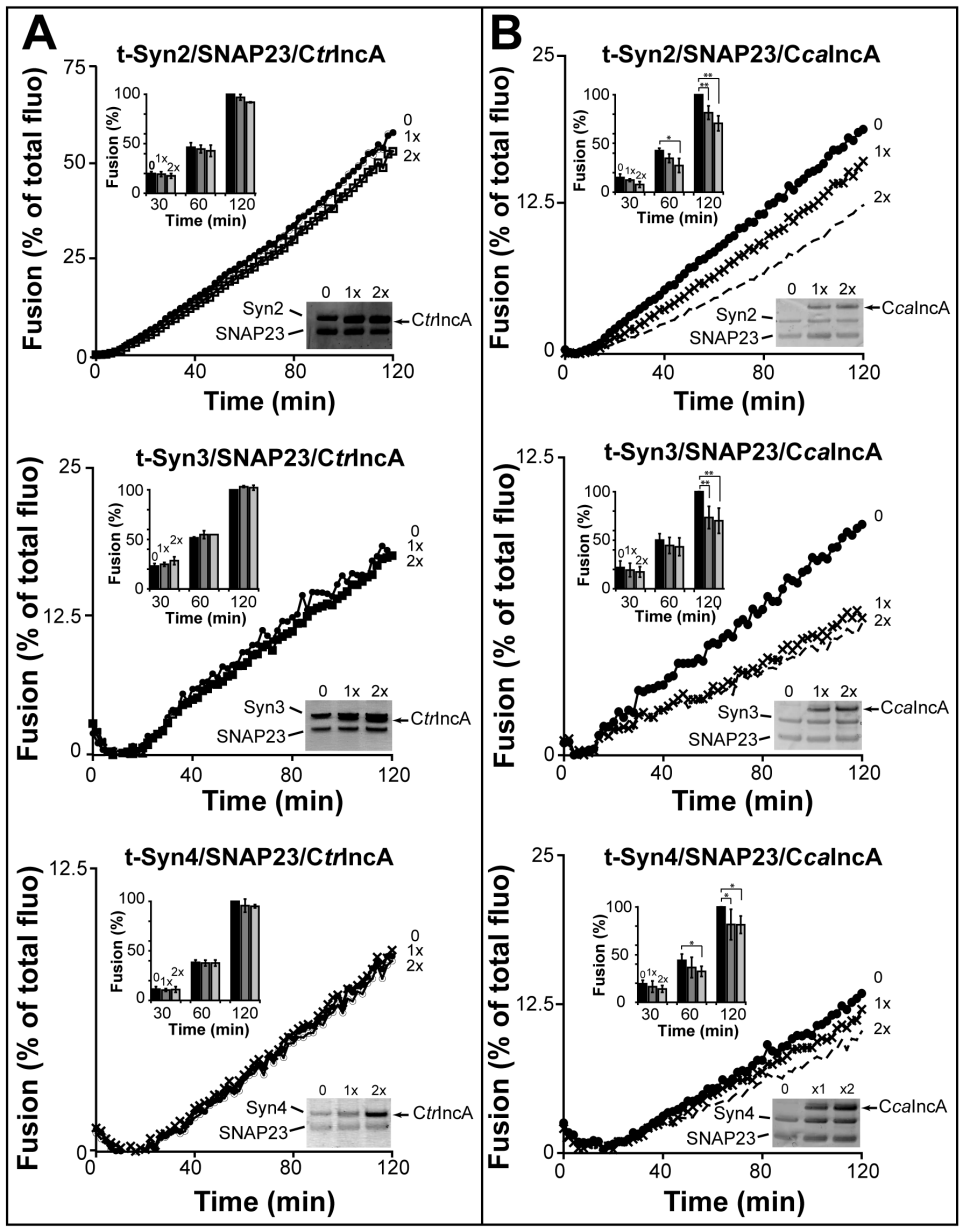
Bacterial SNARE-like proteins display different level of specificity. C*tr*IncA (A) and C*ca*IncA (B) were reconstituted with the exocytic t-SNARE complexes [Syn2/SNAP23], [Syn3/SNAP23] and [Syn4/SNAP23]. After mixing t-SNARE liposomes (with or without IncA) with VAMP2 liposomes, fusion proceeded. Bar graphs represent the mean from n = 5 independent experiments at 30min, 60 min and 120 min for each of the exocytic complex. For the purpose of comparison, maximal values of fusion obtained for the SNARE complex without IncA at 120 min were arbitrarily defined as 100%. The standard deviation is shown. A- As shown on the curves and bar graphs, C*tr*IncA does not affect exocytic fusion regardless of its concentration (p>0.05). B- After 2 hrs of fusion, CcaIncA significantly inhibits [Syn2/SNAP23]-mediated fusion by 35%, [Syn3/SNAP23]-mediated fusion by 25% and [Syn4/SNAP23]-mediated fusion by 20% (p = 0.0079). One and two asterisks denote statistically significant differences with p<0.05, and p<0.02 respectively.

Since *Chlamydia* inclusion membrane is derived from the plasma membrane, we then decided to test the effect of both C*tr*IncA and C*ca*IncA on the plasma membrane resident exocytic t-SNAREs (Table 2). As shown in Fig. 3A, C*tr*IncA has no inhibitory effect on any of the exocytic complexes tested, regardless of its concentration, suggesting that C*tr*IncA is specific for the endocytic SNAREs. C*ca*IncA, on the other hand, exerts a significant inhibitory effect on [Syn2/SNAP23], [Syn3/SNAP23] and [Syn4/SNAP23] fusion (Fig. 3B), demonstrating that C*ca*IncA has a broader inhibitory effect. These results further show that bacterial SNARE-like proteins display different levels of specificity. One might imagine that the capacity to inhibit a large range of membrane fusion events could potentially increase the number of hosts that intracellular bacteria could infect. For example, *C. caviae* has been detected in a wide range of hosts [34]. Alternatively, blocking a multitude of vesicular trafficking in the cells could impact the long-term outcome of an infection. In particular it would be interesting to correlate the level of SNARE-like protein inhibition with the capacity of certain bacteria to induce chronic diseases.

**Figure 4:**
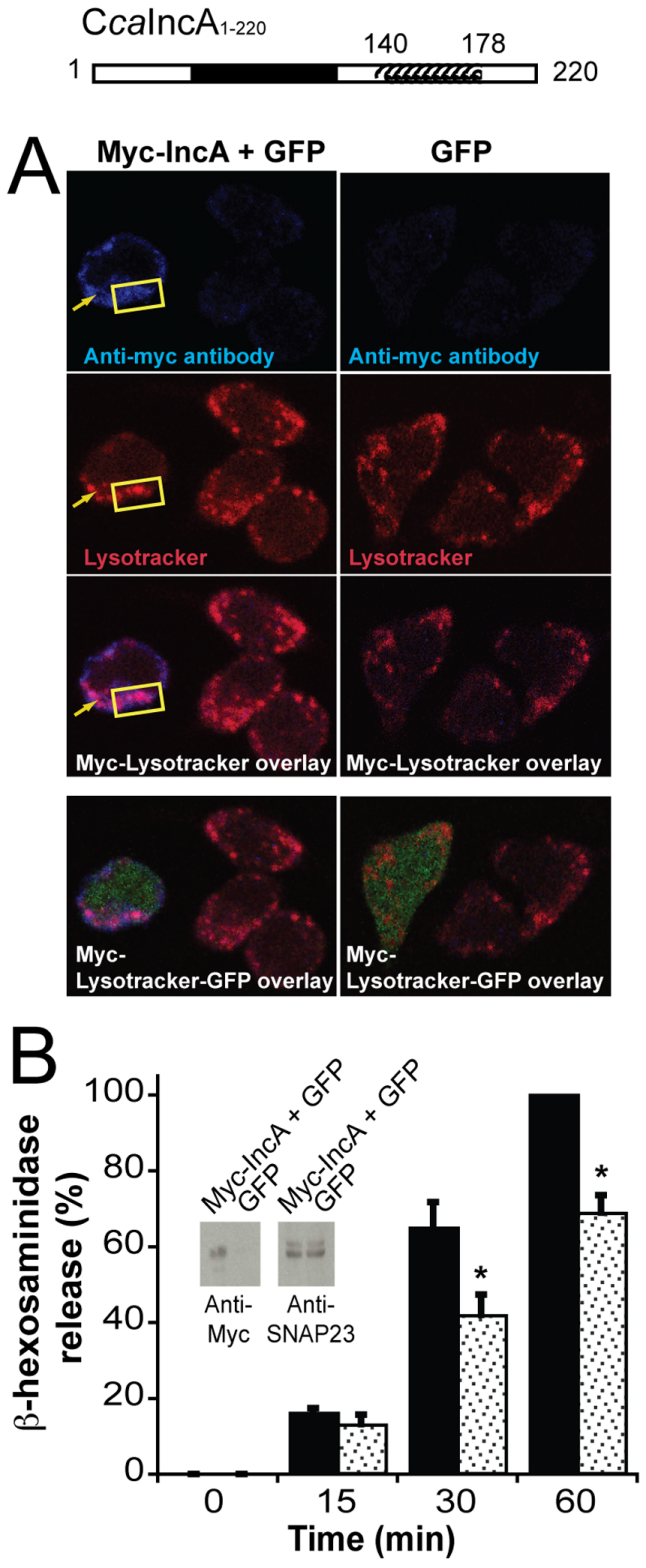
SNARE-like proteins inhibit intracellular fusion in cells. A- Resting transfected RBL-2H3 cells were co-labeled with anti-Myc Abs and lysotracker, and viewed by confocal microscopy. Myc-CcaIncA_1–220_/GFP is on the left, while GFP control is on the right. Co-localized Myc-CcaIncA_1–220_ and lysotracker compartments are indicated with a yellow box and arrows. B-RBL-2H3 cells were transiently transfected with Myc-CcaIncA_1–220_/GFP or with GFP alone. Total lysates were migrated on SDS-PAGE and probed with Abs directed against Myc. Equivalent amounts of protein in each lane was verified after reprobing the blots with the anti-SNAP23. After stimulation of the transfectants at different time points with 10^−7^M PMA/10^−6^M ionomycin, the kinetics of degranulation was analyzed using the β-hexosaminidase release assay. The mean of triplicates from five independent experiments was determined. Standard errors are shown. For the purpose of comparison, maximal values of degranulation obtained for GFP-transfected cells at 60 min were arbitrarily defined as 100%. Transfection of Myc-C*ca*IncA_1–220_ (Grey bars) reduces mast cells degranulation by 23% at 30 min and 31.8% at 60 min compared with GFP (Dark bars). The asterisks denote statistically significant difference (p<0.05) to GFP transfectants. Note that Myc-C*ca*IncA_1–220_/GFP and GFP are not statistically different at 15 min (p = 0.26).

To confirm SNARE-like proteins inhibitory function in a more physiological environment, we tested IncA's role *in vivo* in mammalian cells that can potentially host infection. In order to obtain quantitative results, we chose the RBL-2H3 mast cell line as our model. Mast cells display a large number of endocytic compartments, including their secretory granules, which are secretory lysosomes [35]. During stimulation, the endocytic v-SNAREs VAMP8 present on the secretory lysosomes bind the exocytic t-SNAREs [Syntaxin 4/SNAP23] present on the plasma membrane to mediate exocytosis [36], [37]. If IncA interferes with SNAREs when present in RBL-2H3 mast cells, as it does in the liposomes, we should observe an inhibition of the secretory pathway. Because C*ca*IncA full-length protein was toxic for the cells, RBL-2H3 were transfected with myc-C*ca*IncA_1–220_, a truncated form of C*ca*IncA still containing its SNARE-like N-terminal domain [19], and therefore still inhibitory (see Fig. 2). Myc-C*ca*IncA_1–220_ was cloned together with GFP into an IRES vector to simultaneously express a transfection marker (40% average transfection efficiency). Using immunofluorescence, we observed that Myc-C*ca*IncA_1–220_ co-localized with lysotracker (Fig. 4A), a marker of the RBL-2H3 secretory lysosomes [38]. This suggests that Myc-C*ca*IncA_1–220_ is located on the secretory lysosomes where it can potentially interact with the lysosomal v-SNAREs VAMP8. This is physiologically relevant since VAMP8 is involved in the phagosomal fusion with lysosomes [39]. Therefore, interfering with VAMP8 would protect the phagosomal compartment against degradation. After stimulating transfected mast cells with both 10^−7^M Phorbol 12-Myristate 13-Acetate (PMA) and 10^−6^M ionomycin, we analyzed the release of β-hexosaminidase, a lysosomal enzyme stored inside mast cell secretory lysosomes. Kinetic analyses showed that after 30 min of stimulation, cells transfected with myc-C*ca*IncA_1–220_/GFP secrete significantly less β-hexosaminidase than the GFP control. The level of inhibition at 30 min (23% inhibition) and at 60 min (32% inhibition) is significant (p<0.05 and p<0.02 respectively) compared to GFP transfected cells (Fig. 4B). These data confirm the inhibitory effect of C*ca*IncA on t-[Syn4/SNAP23] and v-[VAMP8]-mediated fusion previously observed using the liposome fusion assay (Figs 3 and 1 respectively). Incidentally, this also indicates that the *in vitro* liposome fusion assay is able to accurately predict cellular data. Therefore, this assay represents a unique system by which more bacterial proteins could be screened for their effect on host vesicular trafficking.

### Conclusion

The key for survival of intracellular bacteria in host cells is their capacity to manipulate host cellular processes -in particular membrane fusion- to allow the establishment of an intracellular replicative niche. An obvious host machinery to target in order to block membrane fusion is the SNARE machinery. Using C*tr*IncA, C*ca*IncA and IcmG/DotF as our models, we demonstrated that SNARE-like bacterial proteins differentially block SNAREs-mediated membrane fusion. Furthermore, we showed that this inhibitory function is encoded into their SNARE-like motifs, validating the general function of such a motif for manipulating membrane fusion.

Interestingly, clinical isolates lacking IncA present defects in their infectious cycle, and the number of inclusions per cells is significantly decreased [40], [41]. This would suggests that the level of protection exerted by *Chlamydia* cell wall during the first 8 hrs [28] is sufficient for small inclusions to develop, but that IncA synthesis is necessary for the inclusions to maturate further. Alternatively, it could also suggest that additional protective systems, although not as efficient IncA, are in place to insure such an important function. Interestingly, *Chlamydia* was found to express additional SNARE-like bacterial proteins, including CT813, which also interacts with host SNARES [21]. Although their inhibitory function remains to be confirmed, the redundancy of the SNARE-like protein system would further support its importance. Overlapping layers of protection would insure the survival of *Chlamydia* in case one of the protective systems fails. The differential timing of expression for each of these proteins could also ensure the protection of the vacuole over time [30]. This redundancy would explain the presence of a limited number of *Chlamydia* inclusions during infections with strains naturally lacking IncA [41]. Each SNARE-like protein may also be specific for a different set of host SNAREs, which would increase the protection of the infectious vacuoles against a larger range of membrane fusion events.

It is intriguing to notice that the inhibitory mechanism displayed by bacterial SNARE-like proteins is very similar to the one employed by the eukaryotic inhibitory-SNAREs (i-SNAREs). SNARE-mediated fusion is triggered by four fusogenic subunits and is highly specific [42], [43], [44], [45]. It was shown that the presence of a fifth SNARE on the same compartment could result in an inhibition of fusion (therefore, such a SNARE has been called inhibitory-SNARE). An i-SNARE can substitute for one of the subunits of the functional tetramer leading to the formation of a non-functional tetramer (acting as a pseudo t-SNARE) [46]. In the Golgi, it has been demonstrated that a gradient of i-SNAREs across cisternae blocks SNARE-mediated membrane fusion and is likely used to fine-tune the specificity of membrane fusion [46]. Here we showed that bacterial SNARE-like proteins appear to function in a similar fashion. Similar to i-SNAREs, these bacterial proteins are capable to bind fusogenic SNAREs and inhibit membrane fusion. Altogether, this suggests that coiled-coil SNARE-like motifs may constitute one of the most effective motifs to manipulate membrane fusion and has been incorporated into intracellular bacteria genome as an adaptation to the pressures of survival [47]. Ultimately, one could take advantage of such a recurrence to develop a common therapeutic strategy for targeting a wide array of bacterial SNARE-like proteins and revert the fusion blockage.

## Materials and Methods

### DNA manipulation and plasmid construction

Standard genetic manipulations were performed throughout. All polymerase chain reaction (PCR) procedures were done with pfu turbo polymerase (Stratagene). All other DNA modifying enzymes were from New England Biolabs. The *E. coli* strain DH5α (Invitrogen) was used for standard cloning. Plasmid encoding C*ca*IncA_1∼22̃_ was generated as described [19]. We added a myc tag and cloned C*ca*IncA_1∼22̃_ into the pIRES2-EGFP vector (Clontech) using the oligonucleotides FO134 GGGAATTCCATATGACAGTATCCACAGACAACAC and FO135 CGGGATCC*TCA*CAGATCCTCTTCTGAGATGAGTTTTTGTTCCAAAGACTGAGCTAATTTCT.

Plasmids encoding Syntaxin 2 (untagged), Syntaxin 3 (untagged), Syntaxin 4 (untagged) and His_6_-SNAP23 were kindly provided by Jingshi Shen (Columbia University, New York). Plasmids encoding Syntaxin 7-His_6_, Syntaxin 8-His_6_, Vti1-His_6_ and VAMP8-His_6_ were generated as described [45]. Plasmids encoding His_6_-C*tr*IncA and His_6_-C*ca*IncA were generated as described in [19]. Plasmid encoding His_6_-IcmG/DotF was generated by PCR using the oligonucleotides FO117 GCGAATTCTCAACTATCTTCTTGACTAAACT and FO118 GGGCATATCCATATGATGGCAGAGCACGATCA. PCR fragments were subsequently ligated into the *EcoRI-NdeI* sites of pET28a. Plasmids encoding His_6_-C*tr*IncA_1–141_, His_6_-C*tr*IncA_1–130_ and His_6_-C*tr*IncA_1–120_ were generated by PCR, respectively using the oligonucleotides FO160 GGGCATATCCATATGACAACGCCTACTCTAATCGTG and FO162 GATGGATCC*CTA*GTCTTTAGATGTCGTTGCAAAT; FO160 and FO163 GATGGATCC*CTA*TAAATGAAGAAATTCTTTCTG. PCR fragments were subsequently ligated into the *NdeI-BamH1* sites of pET28a.

### Protein expression and purification

VAMP8-His_6_, Syntaxin8-His_6_, Syntaxin7-His_6_ and Vti1b-His_6_ were expressed as described [45]. Plasma membrane t-SNARE proteins Syntaxin3/His_6_-SNAP23, Syntaxin4/His_6_-SNAP23, Syntaxin 2/His_6_-SNAP23 were co-expressed in BL21 (DE3) star *E. coli* (Invitrogen) and co-purified using the His_6_ tag present on SNAP23.

All constructs derived from the bacterial proteins: CcaIncA-His_6_, CtrIncA-His_6_, His_6_-C*tr*IncA_1–141_, His_6_-C*tr*IncA_1–130_, His_6_-C*tr*IncA_1–120_ and IcmG/DotF-His_6_ were expressed in BL21 (DE3) star *E. coli* for 12 hrs at 16°C to allow a proper folding of the protein. All his-tagged proteins were purified using the procedure previously described [44], [45], [48].

### Reconstitution into liposomes

SNARE proteins were reconstituted into proteoliposomes by detergent dilution and isolated on an Accudenz density gradient flotation as previously described [6], [49]. To insert bacterial proteins into liposomes, v-SNARE protein and preformed t-SNARE complexes were respectively preincubated with the bacterial protein at different concentration for 4 hrs at 4°C, before being mixed with the lipids, and dialysed for 16 hrs at 4°C.

### Liposome fusion assay

Fusion reactions and data analysis were performed as previously described [6], [49]. For most fusion assays, the mean from at least 5 independent experiments was determined at 30 min, 60 min and 120 min. For the purpose of comparison, maximal values of fusion obtained for the SNARE complex without IncA at 120 min were arbitrarily defined as 100%. The Mann-Whitney *U* test was used to compare the mean values of maximal fusion at 120 min between SNARE-containing liposomes and SNARE/IncA-containing liposomes. Significance was assumed at *p* values<0.05.

### Cell transfection

The rat mast cell line RBL-2H3 was cultured as described [37]. We used the AMAXA nucleofector technology (AMAXA, Germany) to transiently transfect the RBL-2H3 cells. Briefly, 2×10^6^ cells were nucleofected in 100 µl solution V (AMAXA) using 1 µg of pIRES2-EGFP-C*ca*IncA_1∼22̃_ vector or pIRES2-EGFP vector (control). The cells were nucleofected using the program T-030. Cells were then plated in complete medium in 96 well plates for subsequent secretory cell assays 12 hrs later. Using these conditions, the efficiency of transfection was routinely in the range of 30 to 40% as determined by immunofluorescence (GFP positive).

### Confocal immunofluorescence microscopy

Lysotracker labeling was performed following the manufacturer's instruction. Briefly, cells grown on coverslips were incubated with lysotracker 1∶20,000 for 20 min in complete medium and washed three times. The Myc tag labeling was performed as described [37]. We used the anti-myc antibody (9E10) from Santa Cruz Biotechnology. Cy5-conjugated anti-mouse antibody was from Jackson Laboratories. All data were analyzed using a Leica TCS SP confocal microscope, LEICA CONFOCAL 2.5 software, HCX PL APO 63X oil immersion objective.

### Secretory cell assay

Transfectants were plated in 96 well plates in triplicates at ∼5×10^5^ cells in 100 µl of complete DMEM medium and incubated overnight at 37°C. After 12 hrs, adherent RBL cells were washed twice in prewarmed phenol red free DMEM and stimulated by Phorbol Myristate Acetate (10^−7^M)/ionomycin (10^−6^M). At different time points (0, 15 min, 30 min 1 hr), 25 µl of supernatant was collected and the granule secretion marker β-hexosaminidase was analyzed using test supernatants within the linear range of the assay [50]. Total cellular content of β-hexosaminidase was determined by lysis of the adherent cells in 0.5% Triton X-100. The absorbance was determined at 410 nm in a micro-titer plate reader. Results were calculated as a percentage of total β-hexosaminidase in cells after correction for spontaneous release in unstimulated cultures. For the purpose of comparison, all data were normalized to the maximal value of β-hexosaminidase release obtained in pIRES2-EGFP transfectants and arbitrarily taken as 100%. The Mann-Whitney *U* test was used to compare the mean values of maximal release between GFP and Myc-C*ca*IncA _1–220_ transfectants. Significance was assumed at *p* values<0.05.

### SDS-PAGE and Western blot analysis

Western blots were performed as described [51]. The anti-myc antibody (9E10) was from Santa Cruz Biotechnology, the anti-SNAP23 antibody from Synaptic System and both were used at 1∶500. The secondary antibodies were from Biorad and were used at 1∶20,000.
